# Membrane-Lipid Therapy in Operation: The HSP Co-Inducer BGP-15 Activates Stress Signal Transduction Pathways by Remodeling Plasma Membrane Rafts

**DOI:** 10.1371/journal.pone.0028818

**Published:** 2011-12-12

**Authors:** Imre Gombos, Tim Crul, Stefano Piotto, Burcin Güngör, Zsolt Török, Gábor Balogh, Mária Péter, J. Peter Slotte, Federica Campana, Ana-Maria Pilbat, Ákos Hunya, Noémi Tóth, Zsuzsanna Literati-Nagy, László Vígh, Attila Glatz, Mario Brameshuber, Gerhard J. Schütz, Andrea Hevener, Mark A. Febbraio, Ibolya Horváth, László Vígh

**Affiliations:** 1 Institute of Biochemistry, Biological Research Center, Hungarian Academy of Sciences, Szeged, Hungary; 2 Department of Pharmaceutical and Biomedical Sciences, University of Salerno, Salerno, Italy; 3 Department of Biosciences, Abo Akademi University, Turku, Finland; 4 LipidArt Ltd, Szeged, Hungary; 5 Institute of Applied Physics, Vienna University of Technology, Vienna, Austria; 6 Department of Medicine, Division of Endocrinology, University of California, Los Angeles, Los Angeles, California, United States of America; 7 Cellular and Molecular Metabolism Laboratory, Baker Heart and Diabetes Institute, Prahran, Victoria, Australia; University of Arkansas for Medical Sciences, United States of America

## Abstract

Aging and pathophysiological conditions are linked to membrane changes which modulate membrane-controlled molecular switches, causing dysregulated heat shock protein (HSP) expression. HSP co-inducer hydroxylamines such as BGP-15 provide advanced therapeutic candidates for many diseases since they preferentially affect stressed cells and are unlikely have major side effects. In the present study *in vitro* molecular dynamic simulation, experiments with lipid monolayers and *in vivo* ultrasensitive fluorescence microscopy showed that BGP-15 alters the organization of cholesterol-rich membrane domains. Imaging of nanoscopic long-lived platforms using the raft marker glycosylphosphatidylinositol-anchored monomeric green fluorescent protein diffusing in the live Chinese hamster ovary (CHO) cell plasma membrane demonstrated that BGP-15 prevents the transient structural disintegration of rafts induced by fever-type heat stress. Moreover, BGP-15 was able to remodel cholesterol-enriched lipid platforms reminiscent of those observed earlier following non-lethal heat priming or membrane stress, and were shown to be obligate for the generation and transmission of stress signals. BGP-15 activation of HSP expression in B16-F10 mouse melanoma cells involves the Rac1 signaling cascade in accordance with the previous observation that cholesterol affects the targeting of Rac1 to membranes. Finally, in a human embryonic kidney cell line we demonstrate that BGP-15 is able to inhibit the rapid heat shock factor 1 (HSF1) acetylation monitored during the early phase of heat stress, thereby promoting a prolonged duration of HSF1 binding to heat shock elements. Taken together, our results indicate that BGP-15 has the potential to become a new class of pharmaceuticals for use in ‘membrane-lipid therapy’ to combat many various protein-misfolding diseases associated with aging.

## Introduction

A hallmark of stressed cells and organisms is the elevated synthesis of the ubiquitous and highly conserved heat shock protein (HSP) molecular chaperones. The protein quality control and the operation of various stress protection machineries require strict signaling modalities and transcriptional programs which are altered in a number of prominent disease states and, therefore, play a fundamental role in their pathology. Typically, an aberrantly high level of HSPs is characteristic in cancer, and the converse situation applies for aging, type-2 diabetes or neurodegeneration. Understanding those mechanisms whereby mammalian cells can elicit a stress protein response is of key importance and forms the base for the design of new drugs with the ability to modulate the level of a particular HSP [1].

Besides heat shock, many chemicals can induce HSP expression in a temperature- independent manner [2]. Some compounds, including non-toxic hydroxylamine (HA) derivatives [1], [3], may not induce the classical heat shock protein response *per se*, but rather amplify the expression of HSPs induced by mild physical or pathophysiological stresses. Therefore, HSP co-inducers are unique drug candidates because they may enhance HSP expression in diseased cells, without significantly affecting healthy cells [1], [3], [4]. HA derivatives, such as Bimoclomol, Arimoclomol and BRX-220, are effective in the treatment of wound healing in diabetic complications in rats [5], and in delaying the progression of the fatal neurodegenerative condition amyotrophic lateral sclerosis [6]. We have recently shown that another HA derivative NG-094 is remarkably effective at alleviating polyQ-dependent paralysis in *C. elegans* and confers protection against polyQ proteotoxicity even if administered after disease onset, by a mechanism involving HSF1-controlled expression of molecular chaperones [7]. In addition, we have also demonstrated that the HA derivative BGP-15 improves insulin sensitivity in genetic- or diet-induced obesity [8] while others have demonstrated that it protects against tachypacing-induced contractile dysfunction in a *Drosophila* model for atrial fibrillation [9].

There is a growing body of evidence linking the production of HSPs, triggered by exposure to different kinds of environmental stress conditions, to changes in the lipid composition and in the architecture of membranes [10]–[13]. This “membrane sensor” hypothesis predicts that, besides protein denaturation or alteration in nucleic acid conformation, stress protein signals may originate from the cellular membranes [1], [10]. It has been proposed that, rather than the overall changes in the physical state of membranes *per se*, the remodeling of specific microdomains, locally formed nonbilayer structures, and/or changes in the composition of particular lipid molecular species involved directly in specific lipid-protein interactions, are potentially and equally able to furnish stimuli for the activation or attenuation of heat shock genes. A plasma membrane-associated HSP response-refining signal can be related to the altered operation of various membrane-localized receptor proteins, transmitters, lipases or other molecules [14], [15].

Further supporting the view that lipid rafts can be considered to be scaffolds of heat shock response (HSR), the raft-localized sterol glucosyltransferase-driven formation of cholesteryl glucoside (CG), an established HSP-inducing lipid mediator, was shown to be induced before the activation of the heat shock transcription factor (HSF1) [16]. Interestingly, the glycosylation of cholesterol diminishes the ability of the sterol to reside in lateral domains constituted by membrane lipids having highly ordered hydrocarbon chains [17].

In the present study we addressed the mode of action of the HA derivative BGP-15. Hypothesizing that the drug may affect the properties of membranes, by using molecular dynamics (MD) simulations [18] we provided the first evidence on the docking of BGP-15 into model membranes made of sphingomyelin-cholesterol (SM-Chol). The specific interaction of BGP-15 with Chol-containing membranes was further assessed using a combination of complementary biophysical approaches. Chol depletion with methyl-β-cyclodextrin (MBCD) is commonly employed to establish the involvement of lipid rafts in a cellular process. A strongly reduced rate of Chol depletion by MBCD caused by BGP-15 addition was documented *in vitro* by Langmuir monolayer technique. By applying the novel, *in vivo* “thinning out clusters while conserving the stoichiometry of labeling” (TOCCSL) method, which allows imaging of nanoscopic long-lived platforms with raft-like properties diffusing in the live cell plasma membrane [19], we have shown the preservation of raft integrity challenged by mild heat stress in cells pretreated with the HSP co-inducer. Redistribution of Chol-rich membrane domains upon drug administration was followed by a fluorescein ester of polyethylene glycol-derivatized Chol (fPEG-Chol) probe. Inhibition of the small GTPase protein Rac1 [20] demonstrates a profoundly attenuated HSP co-inducing effect of BGP-15. Although the detailed mechanism remains to be elucidated, Rac1 is suggested to be a key element of BGP-15- driven stress signaling pathway upstream of HSF1. We have previously shown that the co-inducing effect of Bimoclomol on HSP expression is mediated via the prolonged activation of HSF1 [21]. Recent studies revealed that the activation of the sirtuin 1 deacetylase (SIRT1) can prolong HSF1 binding to the promoters of heat shock genes by maintaining HSF1 in a more deacetylated, DNA-binding competent state [22]. Thus, we addressed the possible effect of BGP-15 on the regulation of heat-induced acetylation of HSF1. Paradoxically, in 293T cells treated with heat shock alone, an enhanced level of acetylated HSF1 was detected, which returned to baseline during the stress recovery period. BGP-15, however, prevented the heat-induced increase observed in the level of HSF1 acetylation. Finally, we demonstrate that BGP-15 is not a direct modulator of the deacetylase factor SIRT1.

## Results

### BGP-15 partitions into the lipid membranes: molecular dynamics simulation studies

**Figure 1:**
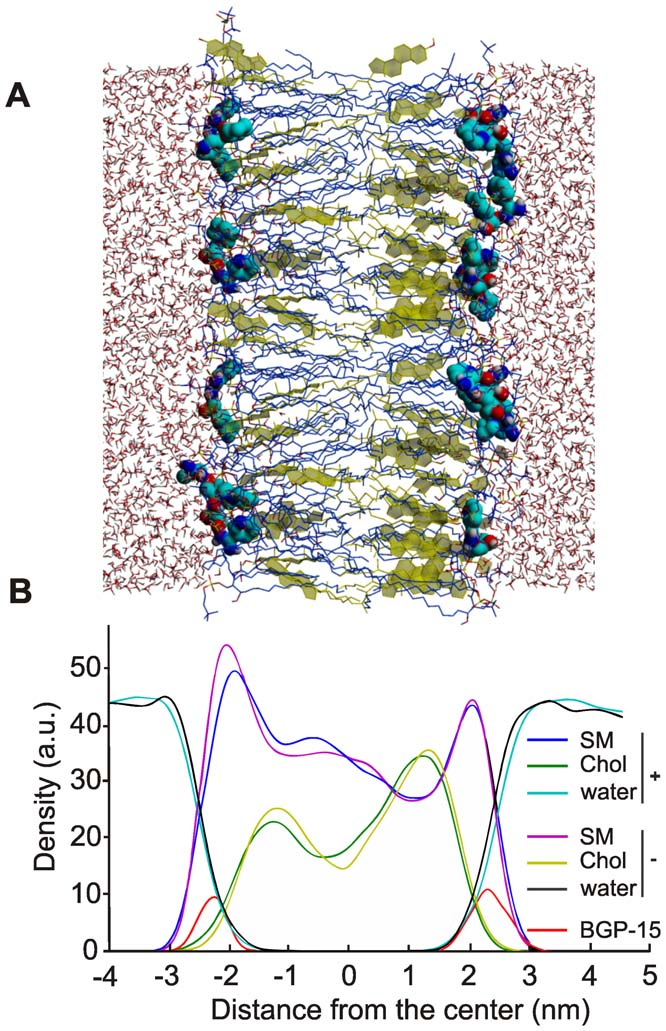
Molecular dynamics simulation of BGP-membrane interaction. **A**) Position of atoms and molecules in SM/Chol membrane after 5 ns of simulation. SM is blue, Chol is yellow, all other atoms of the components and BGP-15 are colored using the Yasara CPK scheme. **B**) The position of BGP-15 molecules (in red) and the structural changes of SM/Chol membranes after addition of BGP-15. The control membrane is indicated with −; the membrane upon addition of BGP-15 is indicated with +.

MD simulations can provide insight into molecular mechanisms associated with the action of a drug and can offer key information about the interactions of a drug with presumptive target biomolecules and have long been used to explore binding, permeation, and accumulation of relevant molecules in membranes [18]. **Figure 1A** represents an MD snapshot of the interaction of the drug with an asymmetric SM/Chol model membrane. **Figure 1B** shows the docking of BGP-15 molecules at the water-lipid interface. The BGP-15 molecules reach the bilayer by diffusion and snorkel just under the level of SM phosphorous in the subsequent 5 ns of simulation. This interaction between the drug and the model membrane modifies the relative spatial positioning of its constituents as highlighted by the changes of the density profile (**Figure 1B**).

### Effect of BGP-15 on the cholesterol depletion in lipid monolayers

**Figure 2:**
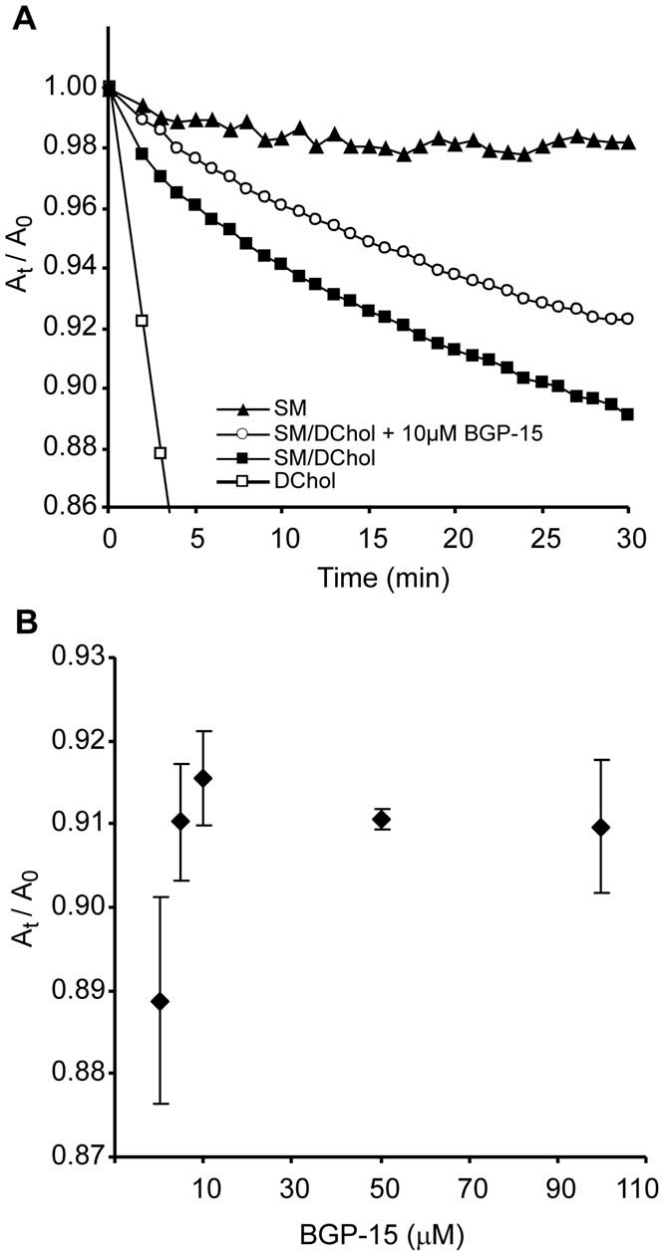
Effect of BGP-15 on DChol desorption from lipid monolayers to MBCD. **A**) MBCD-mediated removal of DChol from lipid monolayers at constant lateral surface pressure. When indicated 10 µM BGP-15 was injected into the subphase underneath SM/DChol monolayer 5 min before MBCD was administered. Surface area was measured before (A_0_) and at the indicated time points after (A_t_) MBCD injection. **B**) Effect of BGP-15 concentration on MBCD-mediated DChol desorption from SM/DChol monolayers. The monolayers were equilibrated with BGP-15 for 5 min before MBCD was injected into the subphase.

Chol release rate is a good measure of the chemical activity of Chol in the membrane [23]. To test whether BGP-15 could have a direct effect on the stability of membrane rafts, we used Langmuir lipid monolayers. The release rate of dihydrocholesterol (DChol) to MBCD from lipid monolayers was measured at constant surface pressure [24]. This technique allows the direct measurement of the removal of DChol from the monolayers by monitoring the decrease in monolayer area. A typical experiment is shown in **Figure 2A**. In the experiments, DChol rather than Chol was used in order to minimize possible artifacts due to Chol oxidation [23]. DChol and Chol have very close chemical structures and DChol is often preferred because of its better stability [25]. Control experiments showed no difference when comparing Chol with DChol. Desorption of DChol from a mixed monolayer containing DChol/SM = 1/1 was markedly slower compared with desorption from pure Chol monolayers (**Figure 2A**). SM removal from the monolayer was negligible since MBCD addition to pure SM monolayer resulted in a very slow decrease in monolayer area. Addition of BGP-15 underneath a DChol/SM = 1/1 monolayer has no effect on the surface pressure (data not shown), however, the desorption of DChol from the monolayer to MBCD decreased at a low 5 µM concentration of BGP-15 in the subphase (**Figure 2B**). This observation suggests that, in the presence of BGP-15 the chemical activity of DChol decreased indicating an increased stability of Chol/SM complexes.

### BGP-15 induces raft remodeling during high temperature stress observed by direct imaging of nanoscopic long-lived rafts in the plasma membrane of live CHO cells

**Figure 3:**
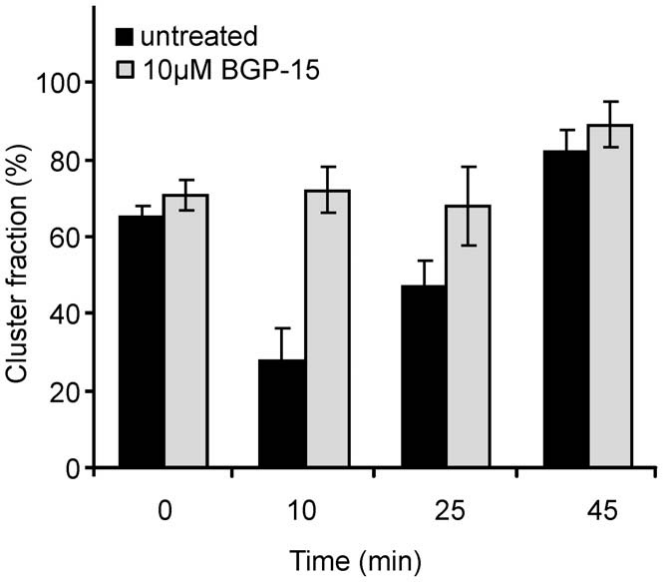
The effect of BGP-15 on the temperature-induced change in cluster fraction of mGFP-GPI. CHO cells stably expressing mGFP-GPI were subjected to 39.5°C with or without 10 µM BGP-15. TOCCSL experiments were performed and at indicated times the cluster fraction (two or more fluorophores per domain) was calculated [n = 2 error bars represent standard error of the mean (SEM)].

While lipid rafts are known to play a crucial role in many signaling processes, relatively little is known regarding their stability, composition or heterogeneity. Importantly, however, activation of the HSR using mild thermal stress coincides with membrane raft reorganization [13], suggesting that the two are directly linked. Recently, we described a novel method, TOCCSL, which allows the direct imaging of nanoscopic long-lived platforms with raft-like properties diffusing in the live cell plasma membrane [19]. Our method senses these platforms by their property to assemble a characteristic set of fluorescent marker proteins or lipids on a time-scale of seconds. In CHO cells stably expressing the consensus raft marker glycosylphosphatidylinositol-anchored monomeric green fluorescent protein (mGFP-GPI), we detected Chol-dependent mGFP-GPI oligomers diffusing as stable units in the plasma membrane [19]. Most importantly, in the course of these studies we also noted that exposure to a mild heat stress (39°C) led to the disappearance of mGFP-GPI homo-association, supporting the idea that the plasma membrane is acutely sensitive to small temperature elevations. We measured the changes in the fraction of mGFP-GPI homo-associates in CHO cells subjected to 39.5°C over a time course of 45 min (**Figure 3**). As expected, the initial cluster fraction dropped to approximately one-third within 10 min at this temperature. BGP-15 pretreatment, however, prevented this early heat-induced raft destabilization indicating that the drug is capable of preserving the integrity of mGFP-GPI-labeled nanoscopic platforms challenged by thermal stress. The spontaneous recovery of raft integrity in control cells observed by the end of the experimental period likely demonstrates the operation of stress-adaptive raft remodeling, as we discussed recently [12], [26].

### BGP-15 affects both the level and the size distribution of cholesterol-rich surface membrane microdomains in heat shock response-defective melanoma cells

When added to live cells, fPEG-Chol is distributed exclusively in the outer plasma membrane leaflet and partitioned preferentially to Chol-rich membrane microdomains [27]. In addition, when mammalian cell number increases beyond a certain level, cells are less stress-inducible for a specific set of HSPs [28].

**Figure 4:**
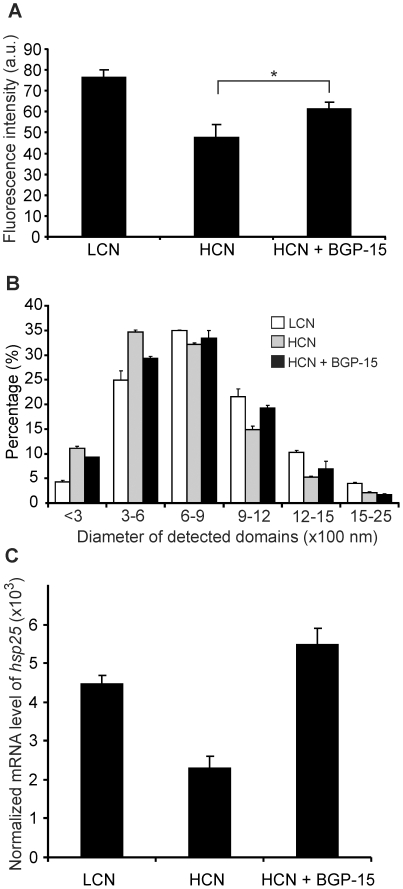
The effect of BGP-15 on cholesterol-rich membrane domains and the heat shock inducibility of *hsp25* in B16-F10 cells with different cell density. **A**) Fluorescence intensity of fPEG-Chol in B16-F10 cells seeded with low (LCN, 1.5×10^6^/10 cm plate) or high cell number (HCN, 6×10^6^/10 cm plate with or without 10 µM BGP-15). Student's t-test was used for statistical analyses, and p = 0.05 was set as a significance threshold. **B**) The size distribution profile of membrane microdomains labeled by fPEG-Chol in HCN (with or without 10 µM BGP-15) and LCN cells and imaged by TIRF microscopy. **C**) HCN (with or without 10 µM BGP-15) and LCN cells were heated at 42°C for 1 hour and the expression of *hsp25* was tested by RT-PCR.

Our results demonstrated that fPEG-Chol is a sensitive and specific probe for unraveling the effect of BGP-15 on the level and size distribution of Chol-rich microdomains in B16-F10 cells. We next assumed that by increasing the chemical activity of Chol, BGP-15 could affect both the level and organization of sterol-rich fPEG-Chol-stained raft domains. According to our model subtle membrane perturbations are critically involved in the conversion of signals from the environment into the transcriptional activation of stress genes. Consistent with this model, when B16-F10 cells were cultured with distinct initial cell densities, we observed marked losses of polyene (especially arachidonic acid) containing lipid molecular species with a concomitant monoene production in conjunction with increasing initial cell number (M.P., G.B., I.H. and L.V. unpublished). Our parallel investigations concerning the inducibility of selected *hsp* genes by RT-PCR also revealed that, together with the lipid molecular species remodeling, such a variation in cell culture conditions seriously affected both the amplitude and profile of HSR (for *hsp25* see later). By using the fPEG-Chol reporter, rafts were imaged by an ultra-sensitive fluorescence microscope in total internal reflection (TIRF) mode and the detected microdomains were ranged in size (diameter) of 300–2500 nm. Most importantly, parallel with the experimentally induced reduction of HSR by increasing the initial cell density in the culture (low initial cell number, LCN: 0.75×10^6^ vs. high initial cell number, HCN: 6×10^6^), the total membrane area covered by fPEG-Chol-positive raft domains decreased by more than 50% (**Figure 4A**) compared with that measured in LCN counterparts. Importantly, however, 60 min pre-treatment with BGP-15 to the HCN sub-population significantly ameliorated this effect (**Figure 4A**). Apart from this effect, administration of BGP-15 also led to a slight alteration in the size distribution of fPEG-Chol-labeled microdomains, separated into six classes on the basis of their diameter (**Figure 4B**). Exclusively in HCN possessing low HS responding capacity, BGP-15 increased the size of membrane rafts at the expense of the smaller ones. Of note, a very similar effect was previously observed following either non-lethal heat priming or after the addition of the HSP inducer benzyl alcohol to B16-F10 cells [13]. In respect of the level and size distribution of membrane microdomains, BGP-15 treatment in HSR-defective HCN melanoma populations resulted in a remarkable similarity to that found in the highly HS-responsive LCN cells. Drug administration rescued the HSR of HCN cells, as highlighted by the two-fold elevation of the expression of *hsp25* gene (**Figure 4C**). Together, these findings suggest that such a drug-induced recovery detected both in the level and size distribution pattern of Chol-rich rafts is very likely coupled to the triggering of raft-associated stress sensing and signaling pathways.

### Rac1 is a key element of BGP-15-driven stress signaling

**Figure 5:**
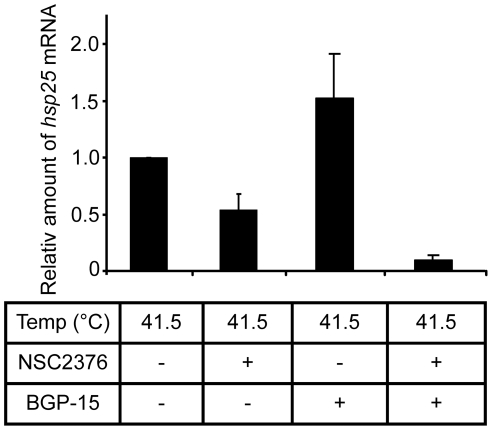
*Hsp25* mRNA expression in B16-F10 cells modified by Rac1 inhibitor and BGP-15 administration. B16-F10 cells were pretreated or not by Rac1 inhibitor NSC23766 for 2 hours before 1 h 41.5°C heat shock with or without 10 µM BGP-15. RNA was isolated and the expression of *hsp25* was tested by RT-PCR. [n = 3, error bars represent standard error of the mean (SEM)].

The Rho family of small GTPases (Rac, Rho, Cdc42) functions as binary switches in various signaling pathways ranged from controlling cell proliferation, differentiation or stress response [20]. Analogous to shear or oxidative stress, moderate heat shock was shown to induce the surface membrane translocation of Rac1 and membrane ruffling in a Rac1-dependent manner, whereas increased HSP expression was paralleled by the activation of HSF1 [29]. Rac1 cycles between the inactive GDP-bound and the active GTP-bound states and its plasma membrane translocation and activation is strongly dependent on membrane Chol content [20]. Integrin signals are known to target Rac1 to Chol-enriched membrane microdomains, where it interacts with downstream effectors [30]. We assumed that since active Rac1 binds preferentially to Chol-rich membranes, and this binding step is specifically determined by the composition and physical state of raft lipids, BGP-15 may affect Rac1 signaling via its interaction with raft constituent lipids. Accordingly, we treated cells with NSC233766, a compound that has been previously shown to specifically inhibit Rac1 GDP/GTP exchange activity by interfering with the interaction between Rac1 and guanine nucleotide exchange factors (GEFs) [31]. Inhibiting Rac1 in this manner diminished heat induced (41.5°C) *hsp25* expression by approximately 50% (**Figure 5**). When cells were heated to 41.5°C and co-treated with BGP-15, the mRNA level of *hsp25* was markedly increased above that of heat treatment alone. Importantly, this effect was blocked by co-treatment with NSC233766 providing clear evidence that the BGP-15-mediated HSP activation is Rac1-dependent. Thus, our data describe a novel mechanism by which HAs, specifically BGP-15, drive stress signaling upstream of HSF1 involving activation of the Rho family member Rac1.

### BGP-15 inhibits HSF1 acetylation monitored during the early phase of heat stress

**Figure 6:**
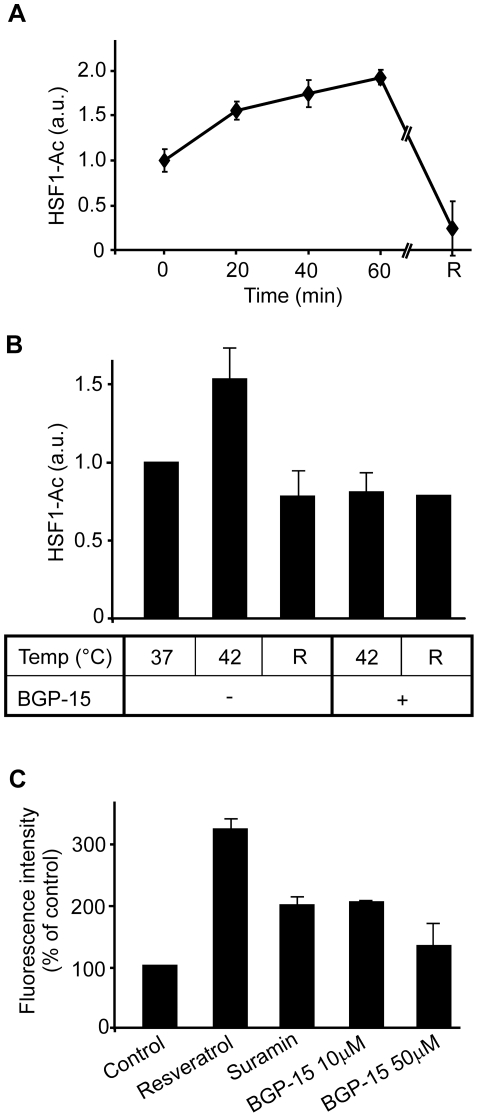
Effect of BGP-15 treatment on heat-induced HSF1 acetylation in HEK293T cells and on *in vitro* SIRT1 activity. **A**) HEK293T cells transiently co-transfected with mouse HSF1-FLAG and p300 were heat shocked for different lengths of time at 42°C or for 60 min at 42°C followed by 60 min recovery (R). After immunoprecipitation by FLAG, samples were probed for acetylated lysin by western blotting [n = 3, error bars represent standard error of the mean (SEM)]; **B**) Samples were or were not treated with 10 µM BGP-15 for 60 min before and during 60 min heat shock or 60 min recovery following heat shock (R) Acetylated HSF1 was determined as above. [n = 4, p<0.05, error bars represent standard error of the mean (SEM)]; **C**) In vitro activity of SIRT1 using activators, inhibitors and BGP-15 [n = 3, error bars represent standard error of the mean (SEM)].

We have previously demonstrated that the HSP co-inducing effect of the HA Bimoclomol is mediated via the prolonged binding of HSF1 to the respective DNA elements [21]. Recently, deacetylation of HSF1 by SIRT1 on Lysine-80 was identified as a novel molecular mechanism resulting in prolonged binding of HSF1 to heat shock element (HSE) [22]. We therefore speculated that administration of BGP-15 might result in a change in the acetylation status of HSF1. To test this hypothesis, a FLAG-tagged mouse HSF1 construct was used to analyze global HSF1 acetylation. Cells exposed to heat shock at 42°C over a 60 min period gradually increased the HSF1 acetylation level compared to untreated (37°C) cells (**Figure 6A**), suggesting paradoxically a stress-triggered attenuation of HSF1-binding on HSE. However, co-treatment with BGP-15 and heat shock for 60 min resulted in a significantly lower acetylated HSF1 level, even when measured immediately after heat exposure (**Figure 6B**).

HSF1 is known to be deacetylated by SIRT1 [22]. To exclude a direct influence of BGP-15 on the SIRT1 enzymatic activity, a commercially available fluorimetric SIRT1-activity assay specifically designed to measure the lysyl deacetylase activity of recombinant SIRT1 was used. In this *in vitro* assay, a peptide comprising amino acids 379–382 of human p53, a known *in vivo* target of SIRT1 activity (Arg-His-Lys-Lys[Ac]), is used as a substrate for a recombinant SIRT1 enzyme. As such, the generated fluorescence signal is proportional to the amount of deacetylation of the lysine corresponding to Lys-382. As expected, the presence of Resveratrol, a known SIRT1 inducer, resulted in an almost three-fold increase while the presence of Suramin, a known SIRT1 inhibitor, resulted in a three-fold lower SIRT1 activity compared with baseline control, respectively. Addition of 10 µM BGP-15 to the reaction resulted in a similar fluorescence signal compared with control, suggesting no direct modulation of SIRT1 activity by BGP-15 (**Figure 6C**). Taken together, BGP-15 prevents the heat-induced immediate acetylation of HSF1 by a hitherto unknown mechanism, rather than via directly affecting the enzyme activity of SIRT1.

## Discussion

For decades, sensing temperature changes in eukaryotes has exclusively been attributed to the formation of unspecified thermolabile proteins, whose unfolding recruits inhibitory chaperones (HSP90, HSC70) in the cytoplasm activating chaperone-repressed HSFs [32]. Recent findings not only allowed a better understanding of the early cellular events that take place at the early phase of temperature rise or other stresses to timely express HSPs, but pointed unambiguously also to membranes as additional cellular sensors in activating a HSR from prokaryotes to mammalian cells [2]. Plasma membrane is not just a barrier separating the cell from its environment but its protein and lipid constituents also coordinate the incoming and outgoing signals [33]. Remodeling of surface membranes initiated either by heat stress or by non-proteotoxic membrane lipid-interacting chemicals can induce generation, transduction, and deactivation of stress signals through global effects on the physical properties (like fluidity) of the membrane matrix [26].

Besides sensing changes in the nanostructure and physical-chemical state of membranes induced directly by stress conditions *per se*, the plasma membrane may also act as a sensor of specific intra- and extracellular protein aggregates, and consequently activate HSP chaperones to reduce aggregate toxicity for membranes [34]. The primary heat stress signals generated in membranes are likely transduced by several transduction pathways [35] and will, however, coalesce predominantly into the activation of HSFs, the expression of HSPs and the onset of cellular thermotolerance [1].

A conclusive picture of HSF1 activation during heat shock and other stresses is yet to be obtained [36]. The activated HSFs would provide the specificity of HSR: the basic stress signal can be as variable as the form of cellular stress stimuli. Aging or pathophysiological conditions can also be linked to the development of subtle membrane changes or “membrane-defects” modulating the operation of such above- described, HSP-refining, membrane-based and/or membrane-controlled “molecular switches”, causing ultimately dysregulated expression of HSPs.

As we defined earlier, a chaperone co-inducer is a substance that cannot induce HSPs by itself, but can enhance HSP induction in combination with other mild stresses. A chaperone co-inducer also has the ability to lower the temperature threshold of the HSR [3]. HAs, such as BGP-15 may provide suitable therapeutic candidates for many disease states since they are capable of affecting stressed rather than unstressed cells and, therefore, they are unlikely to have major side effects as is the case with many classes of current drugs.

The importance of membrane lipids as major therapeutic targets has grown in recent years [37]. This progress is undoubtedly highlighted by the introduction of the concept of “lipid therapy”, in which a drug molecule acts on a membrane to primarily change the membrane structure [38]. Interestingly, the idea of lipid therapy is similar in spirit to the recent concept discussed for the mechanism of general anesthetics [39]. It is noteworthy that anesthetics could play a role in the activation of membrane proteins by changing the pressure profile of a lipid membrane and some of those (like benzyl alcohol) are established HSP inducers [11], [13]. Our studies uniformly indicate that BGP-15 is able to permeate into the surface membranes and to associate prefentially to Chol-enriched lipid platforms. Direct imaging of nanoscopic long-lived platforms with raft-like properties diffusing in the plasma membrane of live mGFP-GPI-transfected CHO cells [19] showed Chol-dependent mGFP-GPI cluster formation. Whereas immediately after exposing cells to temperatures corresponding to fever-stress, the mGFP-GPI clusters almost completely disappeared, while this effect was completely counteracted by the administration of BGP-15. Studies are underway to unravel more precisely how BGP-15 affects the stress-induced raft destabilization/restabilization process, i.e. to participate in the protein and lipid remodeling within membrane nanoplatforms, which is supposed to control the efficacy of the generation and transduction of stress signals towards *hsp* genes.

Next we documented, that the BGP-15-enhanced activation of HSP expression involves the Rac1 signaling cascade in accordance with the previous observation that membrane Chol profoundly affects the targeting of Rac1 to membranes [20]. It was suggested, that stress-stimulated, PI-3-kinase-driven conversion of PIP2 to PIP3 activates the small GTP-binding protein Rac1 under mild, non-denaturing heat stress conditions, which in turn stimulates NADPH oxidase by producing H_2_O_2_ [40]. To explore the importance of signaling mechanisms including lipid peroxidation as a possible subsequent avenue of such a Rac1-dependent oxidative burst coupled with signaling mechanisms affecting HSP expression is presently underway in our laboratory.

Finally, we demonstrate that BGP-15 administration is able to completely inhibit the rapid HSF1 acetylation observed during the early phase of heat stress, thereby very likely promoting a prolonged duration of HSF1 binding to HSE on *hsp* genes [22] as shown first for Bimoclomol [21]. Again, additional studies are needed to elucidate the precise mechanisms through which the drug improves the transcriptional competence of HSF1.

Earlier Csermely and co-workers proposed that drug molecules with multiple targets might have a better chance of affecting the complex equilibrium of whole cellular networks than those acting on a single target. In addition, low-affinity, multi-target drugs were suggested to possess another advantage. Namely, they can increase the number of weak links in preexisting cellular networks and thus stabilize these networks in addition to having multiple effects [41]. In fact, weak links have been shown to stabilize complex networks, including macromolecular networks, and cell membrane is typically such a weak link-controlled macromolecular network. Based on these models, we suggest that BGP-15 is typically a multi-target low-affinity drug. BGP-15 was shown to inhibit the caspase-independent programmed cell death in acetaminophen-induced liver injury [42], and exerted also an effect through modulation of poly(ADP-ribose) polymerase [43]. It is noted, that if multi-target, low-affinity drugs inhibit their targets, they change a strong link into a weak link instead of eliminating the link completely.

Taken together, the non-proteotoxic, lipid-interacting multi-target BGP-15 has a great potential to become a member of a new class of pharmaceuticals for use in ‘membrane-lipid therapy’ to combat most various protein-misfolding diseases and aging.

## Materials and Methods

### Cell culturing

B16-F10 mouse melanoma cell line (American Type Culture Collection, strain C57BL/6J) were cultured in RPMI 1640 medium supplemented with 10% FCS and 4 mM L-glutamine. Human Embryonic Kidney (HEK293T) cells (American Type Culture Collection) were grown in DMEM medium supplemented with 10% FCS. CHO cells stably transfected with mGFP-GPI plasmid [19] were cultured as in [19]. BGP-15 was a gift of N-Gene Research Laboratories, Inc. Budapest, Hungary.

### Molecular modeling

All-atom lipid bilayer model was used as membrane model. The systems studied were asymmetric membranes made of SM: N-octadecanoyl-D-erythro-sphingosylphosphorylcholine and Chol with/without BGP-15 at pH = 7. The SM/Chol membranes were made of 30 SM and 23 Chol molecules in one, and of 19 SM and 26 Chol molecules in the other leaflet. 5 BGP-15 molecules were added on both layers. The simulation box dimensions were X = 101.27 Å, Y = 6.66 Å, and Z = 46.26 Å. 4080 molecules of water to reach the density of 0.997 g/mL have been added. The simulations were carried out with the program YASARA 10.10.28 [44] under NPT ensemble at 310K and 1 atm by coupling the system with a Berendsen thermostat [45] and by controlling the pressure in the manometer pressure control mode. The AMBER03 force field was used. The geometry of the molecules was optimized by semi-empirical PM3 method using the COSMO salvation [46]. Partial atomic charges were calculated using the same level of theory by the Mulliken point charge approach [47]. Electrostatic interactions were calculated with a cutoff of 7.86 Å, and the long-range electrostatic interactions were handled by the Particle Mesh Ewald (PME) [48] algorithm using a sixth-order B-spline interpolation and a grid spacing of 1 Å. The leap-frog algorithm was used in all simulations with a 1.2 fs time step for intramolecular forces and 2.4 fs time step for intermolecular forces and the equilibration period was 5 ns. The lipid bilayers were assembled and relaxed reducing the box dimension till the Van der Waals energy of the system started to increase and the structural parameters of the membranes were comparable to the experimental data [49]. To study the distribution and favorable localization of BGP-15 in a lipid membrane, we applied unconstrained atomistic MD simulations based on passive distribution of probe molecules between bulk water and bilayers. We placed 10 molecules of BGP-15 at random locations and orientations in bulk solution at the vicinity of 5–8 Å from a bilayer surface. To remove high energy contacts, the system was equilibrated at NPT conditions for 5 ns. The membranes were monitored overall the simulation in terms of structural and physical parameters and controlled the thickness, area per lipid and water density adhered to experimental values. The molecules of BGP-15 that were placed at random distances docked to the membrane in the first nanosecond of all simulations.

### Monolayer experiments

Monolayer experiments were carried out with a KSV3000 Langmuir-Blodget instrument (KSV, Finland) in a teflon trough (17 cm×15 cm) at 23°C. Surface pressure was measured by the Wilhelmy method using a platinum plate. Monomolecular lipid layers of egg sphingomyelin (SM) (Larodan, Malmö, Sweden), dihydrocholesterol (DChol) (SUPELCO, Sigma-Aldrich, St. Louis, MO, USA) and SM/DChol = 1∶1 (mol/mol) were spread from chloroform solution (1 mg•mL^−1^) on a PBS subphase (pH 7.2) which was continuously stirred with a magnetic bar. The surface area was held constant until the surface pressure reached an equilibrium after which an initial surface pressure of 22 mN/m) was set and kept constant by changing the monolayer area (barrier speed 4 mm/min). The initial surface pressure is chosen so as to be well above the critical pressure in the phase diagrams of SM/DChol mixtures [23]. BGP-15 (N-Gene, MW = 351,28) was injected under the subphase to get the final concentrations (0, 5, 10, 50, 100 µM ). Methyl β-cyclodextrin (MBCD) (CycloLab, Mw = 1303,4) was added from 100 mM stock in PBS to the subphase to get a final concentrations of 10 mM. Desorption, measured at 22 mN/m was estimated by the area change defined as A_t_/A_0_, where A_0_ and A_t_ are the monolayer area occupied by lipids before and after the injection of MBCD, respectively [25].

### Single molecule microscopy and data analysis

mGFP-GPI-expressing CHO cells were plated onto 30 mm glass slides (#1, Menzel, Braunschweig, Germany) in Petri dishes and incubated overnight in normal growth medium. These slides covered by cells were replaced into POCmini chamber system (LaCon, Staig, Germany) and filled up with preheated growth medium with or without 10 µM BGP-15. Measurements were performed in an accurately thermostated home-built incubator box equipped with a heating unit, a temperable stage insert and an objective heater (Pecon, Erbach, Germany). Measurements were made either at 37 or 39.5°C. Single molecule microscopy setup, measurements and data analysis were described in detail previously [19], [50]. Briefly, a Zeiss Axiovert 200 microscope was equipped with a 100× NA = 1.46 α Plan - APOCHROMAT objective (Zeiss, Oberkochen, Germany). Samples were illuminated in objective-based total internal reflection (TIR) configuration using 488 nm light for excitation. A custom-made program package implemented in LABVIEW (National Instruments, Austin, TX) was used for timing and controlling the microscope setup. Number of fluorescent probes within a single spots was calculated by an in-house algorithms implemented in MATLAB (MathWorks, Natick, MA). Subsequently the percentage of monomer fraction (one mGFP-GPI per a single spot) and cluster fraction (two or more fluorophores per a domain) was determined.

### Experiment with low or high cell number cells

B16-F10 cell were plated at high (HCN = 6×10^6^/10 cm diameter Petri dish) or low (LCN = 0.75×10^6^/10 cm diameter Petri dish) initial cell number.

### Quantitative real-time RT-PCR

Relative quantities of *hsp25* mRNAs were determined and normalized to β-actin as in [13].

### fPEG-Chol labeling

B16-F10 melanoma cells were labeled with fPEG-Chol (kindly provided by T. Kobayashi) and images were taken by CytoScout fluorescent microscope. 200 nM fPEG-cholesterol (kindly provided by T. Kobayashi) labeling solution was prepared in PBS. B16-F10 cells were grown in glass-bottom dishes with two different initial cell densities: low initial cell number, LCN: 0.75×10^6^ and high initial cell number, HCN: 6×10^6^. In the case of BGP-15 pretreatment 10 µM BGP-15 was added to cells before the labeling for 20 min. Samples were labeled with the labeling solution for 15 min at room temperature. After washing fresh RPMI without phenol-red was added to cells and images were taken by CytoScout fluorescent microscope. Samples were illuminated in objective-based total internal reflection (TIR) configuration using 488 nm light for excitation, images were analyzed in CellProfiler (www.CellProfiler.org) and ImageJ software (http://rsbweb.nih.gov/ij/). Both the mean fluorescent intensity and the size (theoretical diameter) of each single detected microdomain were calculated. The domains we found were sorted into classes according to theoretical diameter. Diameters were calculated as 2X * (Npix/π)-2, where X is the size of a pixel in nm and Npix is the number of pixels covering the actual domain.

### Rac1 inhibitor assay

B16-F10 cells were treated with 100 µM Rac1-specific inhibitor, NSC23766 (Santa Cruz Biotechnology, USA) for 2 hours. It was followed by 1 hour heat shock at 41.5°C along with/without 10 µM of BGP-15. After overnight recovery at 37°C, cell lysates were prepared in Laemmli Buffer. Equal amounts of proteins were loaded to 15% SDS gel and transferred to PVDF membrane. Membranes were probed with anti HSP25 antibody (SPA 801), Stressgen). Expression levels were visualized with Enhanced Chemiluminescence (Amersham, USA). Band intensities were measured with Alpha View Software v.1.3.0.7. (Alpha Innotech, USA) and normalized to 41.5°C heat-treated expression level of HSP25.

### HSF1 acetylation assay

HEK293T cells were transiently co-transfected with mouse HSF1-FLAG and p300 (gift of S. Westerheide) as in [22]. 48 hours after transfection, cells were preincubated for 1 hour with 10 µM BGP-15 after which 1 hour heat shock at 42°C was applied. Immediately after heat shock or after one hour recovery at 37°C, cell lysates were prepared in standard RIPA buffer and HSF1-FLAG was pulled down overnight at 4°C with anti-FLAG M2 affinity gel beads (Sigma, F2426). Acetylated-HSF1 or total FLAG was determined by western blotting with an anti-acetylated-lysine antibody (Cell Signaling, #9441) or anti-FLAG M2 antibody (Sigma, F1804), respectively. Expression levels were determined as above for HSP25. Band intensities were expressed as the ratio of acetylated-HSF1 over FLAG as obtained in the same pull down reaction and the ratio obtained at 37°C was considered as 1. Data are expressed as mean and standard error of the mean (SEM).

### Fluor de Lys SIRT1-deacetylase assay

*Fluor-de Lys* deacetylase assay kit (Biomol International, USA) was used to analyze the direct effect of BGP-15 on the deacetylase activity of recombinant SIRT1 according to the manual. Nicotinamide was added to the reaction at the concentration of 2 mM when necessary. The final fluorescence was measured in a Fluoroskan Ascent FL fluorometer (Thermo Fisher Scientific, USA).
